# Dysentery

**Published:** 1888-02

**Authors:** S. H. Cowden

**Affiliations:** Solgohacie, Ark.


### DYSENTERY.

Editor Daniel's Texas Medical Journal.

I am much pleased with your Journal, and see in it a "ready reference" for the busy practitioner.

The object of this short note is to suggest to your readers the use of the following enema in dysentery. When the stools are fœtid and contain blood, this receipt will quickly change their character, and relieve the tormina and tenesmus. It is as follows:

—Receipt.—Mucil, Amyli.................................℥ii
Pulv. Ipecac..............................xx gr.
Morph. Acet..............................¼, ½ grs.

Misce.

The patient should be placed on the left side, and the rectal tube iusertcd. Inject one-half the enema, and turn the patient on the back and inject the remaining portion. It has proved, in my hands, signally beneficial. It is also beneficial in the dysentery which sometimes attends the puerperal state.

S. H. COWDEN, M. D.
Solgohacie, Ark.

